# Angiotensin-[1–7] attenuates kidney injury in experimental Alport syndrome

**DOI:** 10.1038/s41598-020-61250-5

**Published:** 2020-03-06

**Authors:** Hong Sang Choi, In Jin Kim, Chang Seong Kim, Seong Kwon Ma, James W. Scholey, Soo Wan Kim, Eun Hui Bae

**Affiliations:** 1 Departments of Internal Medicine, Chonnam National University Medical School, Gwangju, Korea; 2 Department of Medicine and Institute of Medical Science, University of Toronto, Toronto, Canada

**Keywords:** Molecular medicine, Target identification

## Abstract

Angiotensin-[1–7] (Ang-[1–7]) antagonize the actions of the renin-angiotensin-system via the Mas receptor and thereby exert renoprotective effects. Murine recombinant angiotensin-converting enzyme (ACE)2 was reported to show renoprotective effects in an experimental Alport syndrome model; however, the protective effect of direct administration of Ang-[1–7] is unknown. Here, we used *Col4a3*^−/−^ mice as a model of Alport syndrome, which were treated with saline or Ang- [1–7]; saline-treated wild-type mice were used as a control group. The mice were continuously infused with saline or Ang-[1–7] (25 μg/kg/h) using osmotic mini-pumps. *Col4a3*^−/−^ mice showed increased α-smooth muscle actin (SMA), collagen, and fibronectin expression levels, which were attenuated by Ang-[1–7] treatment. Moreover, Ang-[1–7] alleviated activation of transforming growth factor-β/Smad signaling, and attenuated the protein expression of ED-1 and heme oxygenase-1, indicating reduction of renal inflammation. Ang-[1–7] treatment further reduced the expression levels of inflammatory cytokines and adhesion molecules and attenuated apoptosis in human kidney cells. Finally, Ang-[1–7] downregulated TNF-α converting enzyme and upregulated ACE2 expression. Thus, treatment with Ang-[1–7] altered the ACE2-Ang-[1–7]-Mas receptor axis in the kidneys of *Col4a3*^−/−^ mice to attenuate the nephropathy progression of Alport syndrome.

## Introduction

The renin-angiotensin system (RAS) plays a critical role in the development and progression of kidney diseases^1^. Thus, blockade of RAS, including the use of angiotensin-converting enzyme (ACE) inhibitors or angiotensin II receptor blockers (ARB), can effectively suppress the progression of kidney diseases in both animal experiments and large-scale clinical studies. Accordingly, ACE inhibitors or ARBs are currently recommended as first-line therapy for renoprotection in non-diabetic and diabetic patients with chronic kidney disease (CKD)^2^. The ACE2/angiotensin-[1–7] [Ang-[1–7]]/Mas receptor axis plays a counter-regulatory role to the ACE/Ang II/Ang II type 1 receptor (AT1R) axis of the RAS, and its activation has been shown to exert a renoprotective effect in kidney diseases^3,4^. However, its influence on protection against progressive kidney disease in the context of Alport syndrome (AS) has not been evaluated to date.

AS is a hereditary nephropathy characterized by progressive kidney disease and hearing loss, which is caused by mutations in the genes encoding the alpha 3, alpha 4, or alpha 5 chain comprising the type IV collagen that forms the glomerular basement membrane, leading to glomerulosclerosis^5,6^. Renal problems begin with asymptomatic hematuria, followed by progressive proteinuria, and eventually CKD. Treatment with RAS blockers has shown good effects in slowing down the rate of deterioration of kidney disease in both experimental models and clinical trials of patients with AS^7–10^, leading to the recommendation of their use in the clinical management of AS^11^. Mice with the *Col4a3* gene knocked out (*Col4a3*^−/−^) thus serve as a reliable experimental model of AS and CKD. In the *Col4a3*^−/−^ mice, microscopic hematuria and proteinuria are observed. As CKD progresses, end-stage kidney disease develops and the mean age of death is 14 weeks^12^. A previous study showed an increase in Ang II and decrease in Ang-[1–7] levels in the kidneys of *Col4a3*^−/−^ mice, suggesting a potential pathogenic role in the renal damage in AS^13^. Moreover, the activity of intrarenal ACE2 in *Col4a3*^−/−^ mice was decreased and the progression of kidney injury was significantly inhibited by administration of murine recombinant ACE2^14^, which was accompanied by an increase in intrarenal Ang-[1–7] expression, suggesting that administration of Ang-[1–7] may also have a renoprotective effect similar to ACE2.

Thus, to better understand the role of the ACE2/Ang-[1–7]/Mas receptor axis in AS, we treated *Col4a3*^−/−^ mice as a common model of AS with Ang-[1–7], and evaluated the direct effects of Ang-[1–7] infusion on the progression of kidney injury in AS. Since 80% of AS patients are known to be X-linked and more severe phenotypes in male^15^, only male mice were used for our study. Overall, our findings demonstrate the potential of Ang-[1–7] to attenuate inflammation, apoptosis, and renal injury in AS, as a candidate target for development of new treatment strategies.

## Results

### Ang-[1–7] attenuates morphological changes in experimental AS

**Figure 1:**
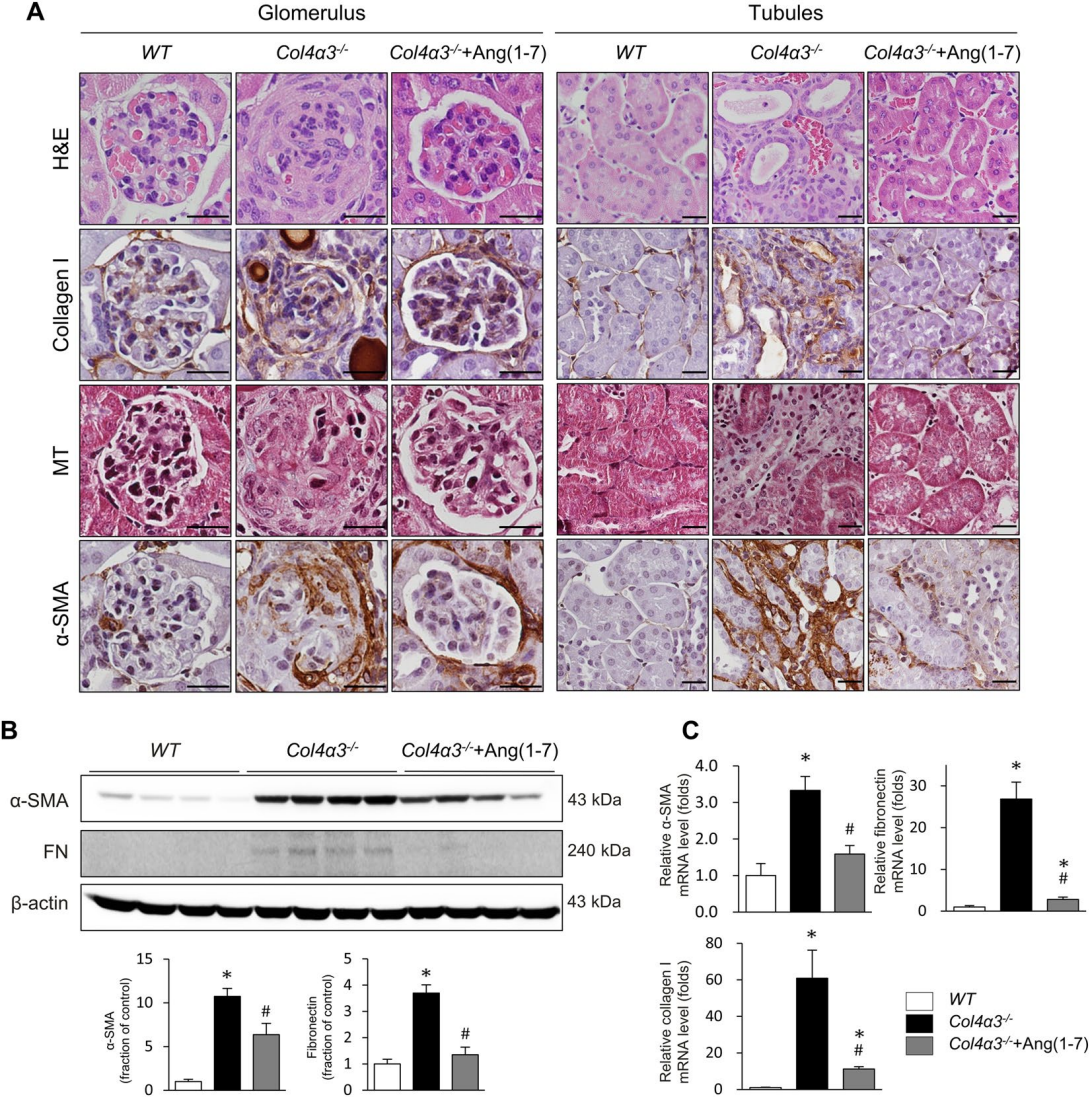
Effects of Ang-[1–7] on kidney fibrosis in *Col4a3*^−/−^ mice kidneys. (**A**) Tissue morphology of kidney from WT, *Col4a3*^−/−^, and *Col4a3*^−/−^+Ang-[1–7] mice. Images from glomerulus (left) and tubulointerstitium (right) are presented. (**B**,**C**) Comparison of expression level for fibrosis markers determined by immunoblotting (**B**) and qPCR (**C**) from the kidney of WT, *Col4a3*^−/−^, and Col4a3^−/−^+Ang-[1–7] mice (n = 4 mice/group). Scale bars, 25 μm. H&E, hematoxylin & eosin staining; MT, Masson’s trichrome staining; α-SMA, alpha smooth muscle actin; FN, fibronectin.

At 7 weeks of age, *Col4a3*^−/−^ mice showed a considerably higher urine albumin-to-creatinine ratio (ACR) than wild-type (WT) mice (Table 1). Although the differences did not reach statistical significance, there was a marked numerical decrease in the urine ACR after treatment with Ang-[1–7]. The levels of urinary neutrophil gelatinase-associated lipocalin (NGAL) excretion, as a marker of tubular injury, were significantly higher in *Col4a3*^−/−^ mice than those of WT mice, but were reduced by Ang-[1–7] treatment. Even after normalization of NGAL level to urinary creatinine, significant changes were observed consistently. Hematoxylin and eosin (H&E) staining in the kidney sections revealed glomerular sclerosis and interstitial infiltration of mononuclear cells in *Col4a3*^−/−^ mice relative to those of WT mice (Fig. 1A). However, these changes were clearly attenuated by Ang-[1–7] treatment. Further, Masson’s trichrome staining showed deposition of interstitial collagen in the kidneys of *Col4a3*^−/−^ mice, which was also attenuated by Ang-[1–7] treatment. Immunohistochemical staining revealed the increased accumulation of type I collagen in the peritubular and periglomerular interstitium in the kidneys of *Col4a3*^−/−^ mice, which was attenuated by Ang-[1–7] treatment (Fig. 1A).

**Table 1:** Effect of Ang-[1–7] on the renal function in *Col4a3*^−/−^ mice.

	*WT*	*Col4a3*^−/−^	C*ol4a3*^−/−^+Ang-[1–7]
Body weight (g)	22.6 ± 1.7	21.6 ± 1.0	22.6 ± 0.6
Kidney weight (g)	0.15 ± 0.01	0.18 ± 0.01	0.19 ± 0.01*
Kidney weight/body weight (g/kg)	6.6 ± 0.3	8.5 ± 0.3*	8.3 ± 0.4*
Urine creatinine (mg/dl)	61.76 ± 12.47	24.99 ± 8.53	35.20 ± 6.68
Urine albumin (μg/ml)	4.00 ± 0.00	115.87 ± 12.50*	97.76 ± 30.21*
Urine albumin-to-creatinine ratio (mg/g Cr)	7.46 ± 1.26	784.00 ± 247.16*	389.46 ± 169.71
Urine NGAL (ng/ml)	77.80 ± 10.86	1616.27 ± 64.55*	401.60 ± 137.74^#^
Urine NGAL-to-creatinine ratio (ng/mg Cr)	136.60 ± 15.51	10057.85 ± 2579.52*	1085.15 ± 257.68*^#^

Abbreviations: WT, wild-type; ang-[1–7], angiotensin-[1–7]; NGAL, neutrophil gelatinase-associated lipocalin.

^*^p < 0.05 compared with WT. ^#^p < 0.05 compared with *Col4a3*^−/−^. Values are expressed as the mean ± SEM.

### Ang-[1–7] ameliorates kidney fibrosis in experimental AS

To investigate the effects of Ang-[1–7] on kidney fibrosis, expression levels of the myofibroblast molecular marker alpha-smooth muscle actin (α-SMA) and fibronectin were compared among the groups. Immunoblotting of α-SMA and fibronectin showed that the expression of both profibrotic markers was considerably up-regulated in the kidneys of *Col4a3*^−/−^ mice, which was prevented by Ang-[1–7] treatment (Fig. 1B). Quantitative polymerase chain reaction (qPCR) confirmed the significant upregulation of the mRNA expression of α-SMA, fibronectin, and collagen I in the AS model, and these changes were attenuated by Ang-[1–7] treatment (Fig. 1C). Immunohistochemical staining for α-SMA revealed its increased expression in the peritubular and periglomerular interstitium in *Col4a3*^−/−^ mouse kidneys, which was significantly reduced by Ang-[1–7] treatment (Fig. 1A).

### Ang-[1–7] inhibits transforming growth factor (TGF)-β/Smad signaling activation in experimental AS

**Figure 2:**
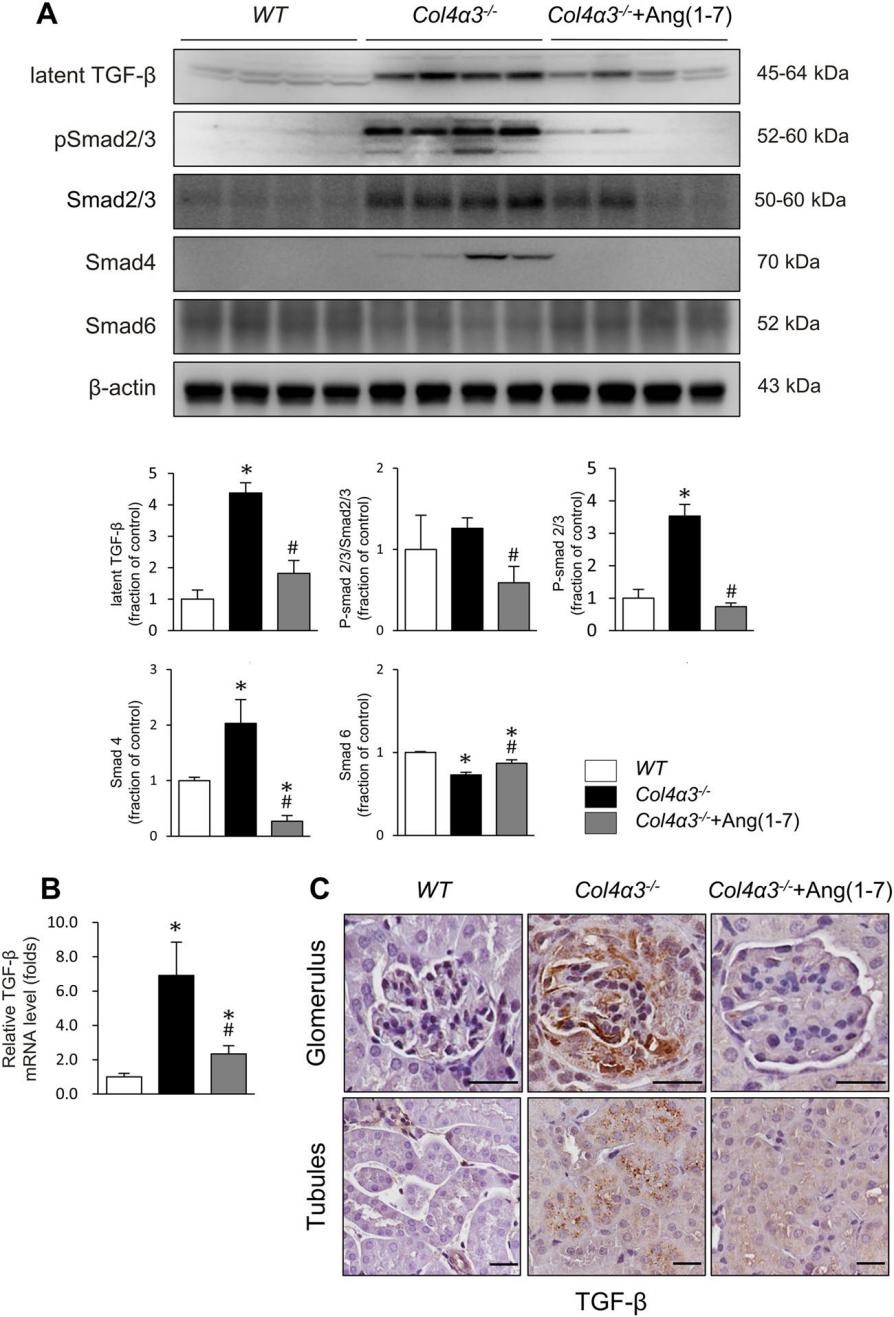
Effects of Ang-[1–7] on transforming growth factor-beta (TGF-β)/Smad pathway in *Col4a3*^−/−^ mice kidneys. (**A**) Protein expression of the TGF-β and Smad proteins was assessed in kidney of WT, *Col4a3*^−/−^, and *Col4a3*^−/−^+Ang-[1–7] mice (n = 4 mice/group). β-actin was used as the endogenous control. (**B**) Comparison of mRNA expression level TGFβ determined by qPCR from the kidney WT, *Col4a3*^−/−^, and *Col4a3*^−/−^+Ang-[1–7] mice (n = 4 mice/group). (**C**) Representative immunohistochemical staining of TGF-β in renal cortex of WT, *Col4a3*^−/−^, and *Col4a3*^−/−^+Ang- [1–7] mice. Each column represents mean ± SEM. *P < 0.05 vs. WT mice; ^#^P < 0.05 vs^.^ *Col4a3*^−/−^ mice.

To elucidate the signaling pathway related to renal fibrosis occurring in the kidneys of *Col4a3*^−/−^ mice, we performed an immunoblot assay, qPCR, and immunohistochemical staining on the factors involved in the TGF-β/Smad pathway, as a critical mediator of renal fibrosis. In the immunoblot and qPCR analyses, the protein and mRNA expression levels of latent TGF-β1 were considerably increased in the kidneys of *Col4a3*^−/−^ mice (Fig. 2A,B). Further, phosphorylation of the downstream signal mediator signal Smad2/3 showed trend to the increasing expression level and expression level of Smad4 was also increased in the *Col4a3*^−/−^ mouse kidneys, demonstrating canonical TGF-β signaling pathway activation (Fig. 2A). However, with Ang-[1–7] treatment, the expression levels of latent TGF-β1, phosphorylated Smad2/3, and Smad4 were significantly reduced compared with those of untreated *Col4a3*^−/−^ mice. By contrast, the expression level of Smad6, an inhibitory factor of Smad that negatively regulates TGF-β/Smad signaling, was decreased in the *Col4a3*^−/−^ mouse kidneys, which was recovered by Ang-[1–7] treatment (Fig. 2A). Immunohistochemical staining revealed the increased expression of TGF-β in *Col4a3*^−/−^ mouse kidneys, which was attenuated by Ang-[1–7] treatment (Fig. 2C). These results confirmed that TGF-β/Smad signaling activation is involved in the occurrence of renal fibrosis in *Col4a3*^−/−^ mice, which can be reversed with Ang-[1–7] treatment.

### Ang-[1–7] attenuates inflammation and apoptosis in experimental AS

**Figure 3:**
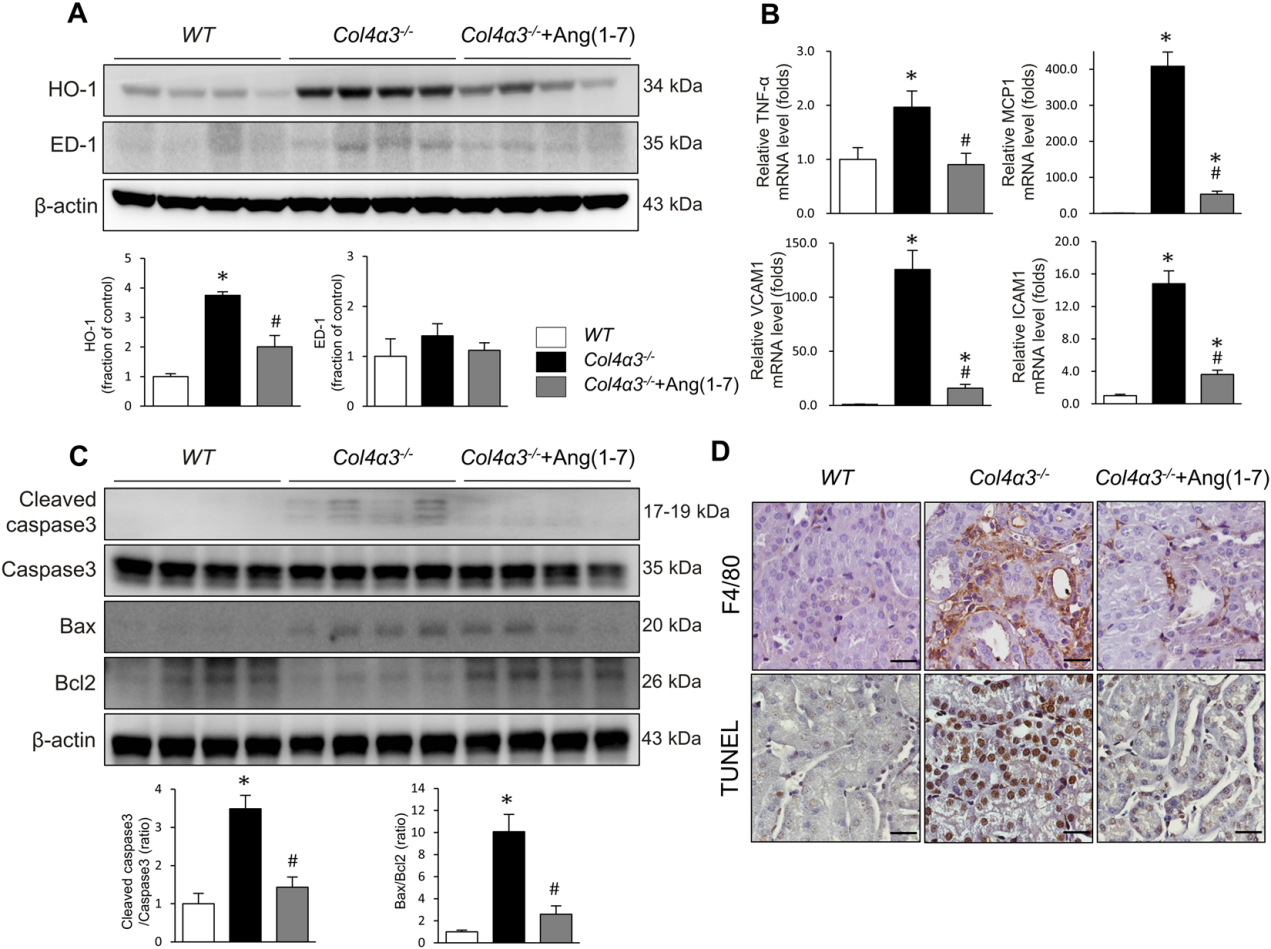
Effects of Ang-[1–7] on inflammation and apoptosis in *Col4a3*^−/−^ mice kidneys. (**A**) Comparison of protein expression level for HO-1 and ED-1 determined by immunoblotting from the kidney of WT, *Col4a3*^−/−^, and *Col4a3*^−/−^+Ang-[1–7] mice (n = 4 mice/group). β-actin was used as the endogenous control. (**B**) Comparison of mRNA expression level for inflammatory markers determined by qPCR from the kidney of WT, *Col4a3*^−/−^, and *Col4a3*^−/−^+Ang-[1–7] mice (n = 4 mice/group). (**C**) Comparison of protein expression level for proteins related to apoptosis determined by immunoblotting from the kidney of WT, *Col4a3*^−/−^, and *Col4a3*^−/−^+Ang-[1–7] mice (n = 4 mice/group). β-actin was used as the endogenous control. (**D**) Representative images of immunohistochemical staining for F4/80 (upper) and TUNEL (lower) in the kidney of WT, *Col4a3*^−/−^, and *Col4a3*^−/−^+Ang-[1–7] mice. Scale bars, 25 μm. Each column represents mean ± SEM. *P < 0.05 vs. WT mice; ^#^P < 0.05 vs^.^ *Col4a3*^−/−^ mice. MCP-1, monocyte chemoattractant protein-1; TNF-α tumor necrosis factor-α; ICAM-1, intercellular adhesion molecule-1; VCAM-1, vascular cell adhesion molecule-1.

We next examined the expression level of heme oxygenase-1 (HO-1) by immunoblot analysis to assess the degree of oxidative stress in the kidney. We found increased expression levels of HO-1 in the kidneys of *Col4a3*^−/−^ mice, which was attenuated by Ang-[1–7] treatment (Fig. 3A). However, the levels of ED-1, a marker of tissue inflammation, were not different between the three groups of mice (Fig. 3A), despite evidence of active inflammation in the kidneys of *Col4a3*^−/−^ mice as indicated by higher transcript levels of proinflammatory cytokines and adhesion molecules in qPCR analyses. Specifically, the mRNA expression levels of monocyte chemoattractant protein (MCP)-1, TNF-α, ICAM-1, and VCAM-1 were significantly increased in the kidneys of *Col4a3*^−/−^ mice, and administration of Ang-[1–7] considerably suppressed the increase of these molecules (Fig. 3B). Immunohistochemical staining for F4/80, a marker of murine macrophage populations, revealed its increased expression in the interstitial space of the kidneys of *Col4a3*^−/−^ mice, which was also attenuated by Ang-[1–7] treatment (Fig. 3D). Taken together, these data suggest that Ang-[1–7] suppresses renal inflammation and oxidative stress in *Col4a3*^−/−^ mice.

To evaluate the degree of cell death of the renal tubular cells in *Col4a3*^−/−^ mice, the alterations of apoptotic proteins were assessed. Immunoblotting revealed an increased BAX/BCL-2 ratio and cleaved caspase 3/caspase 3 ratio in the kidney sections of *Col4a3*^−/−^ mice, suggesting that apoptosis was exacerbated (Fig. 3C). These changes were not observed in the mice treated with Ang-[1–7]. Terminal deoxynucleotidyl transferase dUDP nick-end labeling (TUNEL) further demonstrated a higher number of TUNEL-positive tubular epithelial cells in the cortex of the kidneys of *Col4a3*^−/−^ mice compared to that of control mice (Fig. 3D), whereas Ang-[1–7] treatment decreased the numbers of TUNEL-positive cells. Indeed, Ang-[1–7] treatment clearly reduced apoptotic process in induced by the lack of *Col4a3* in the kidney cells.

### Ang-[1–7] reverses the downregulation of ACE2 in *Col4a3*^−/−^ mice

**Figure 4:**
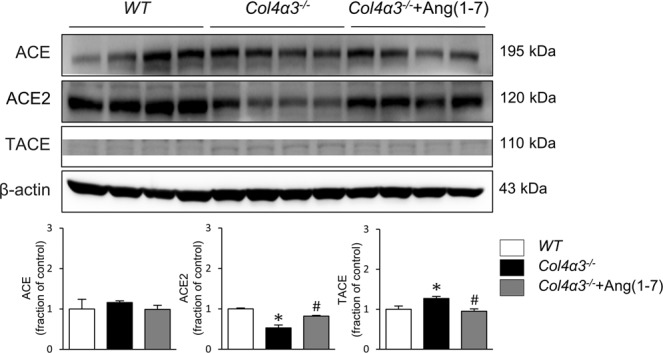
Effects of Ang-[1–7] on renin angiotensin system (RAS) in *Col4a3*^−/−^ mice kidneys. Comparison of protein expression level of RAS components determined by immunoblotting from the kidney of WT, *Col4a3*^−/−^, and *Col4a3*^−/−^+Ang-[1–7] mice (n = 4 mice/group). β-actin was used as the endogenous control. Each column represents mean ± SEM. *P < 0.05 vs. WT mice; ^#^P < 0.05 vs^.^ *Col4a3*^−/−^ mice. ACE, angiotensin converting enzyme; TACE, TNF-α converting enzyme.

**Figure 5:**
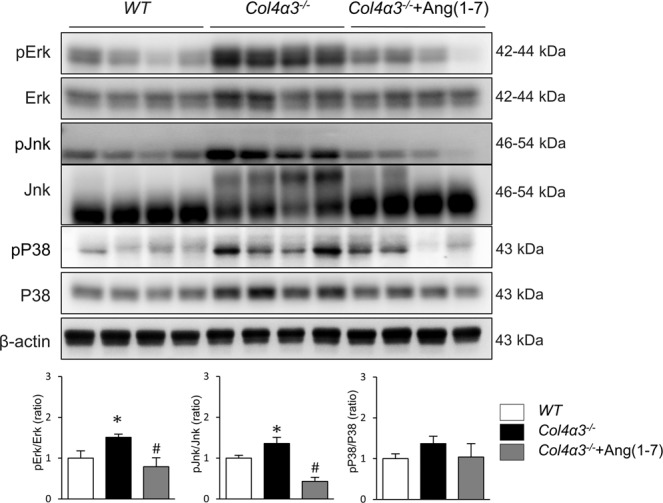
Effects of Ang-[1–7] on mitogen-activated protein kinase (MAPK) pathway in *Col4a3*^−/−^ mice kidneys. Protein expression of the total ERK, phosphorylated ERK (p-ERK), total JNK, phosphorylated JNK (p-JNK), P38 and phosphorylated P38 (pP38) was assessed in the kidney of WT, *Col4a3*^−/−^, and *Col4a3*^−/−^+Ang-[1–7] mice (n = 4 mice/group). β-actin was used as the endogenous control. Each column represents mean ± SEM. *P < 0.05 vs. WT mice; ^#^P < 0.05 vs^.^ *Col4a3*^−/−^ mice.

Based on the results described above, we hypothesized that the intrarenal RAS plays a role in regulating the upstream signals that induce renal fibrosis, inflammation, and apoptosis in *Col4a3*^−/−^ mice, which were all ameliorated by Ang-[1–7]. As shown in Fig. 4, there was no difference in the expression levels of ACE protein between the kidneys of *Col4a3*^−/−^ mice and WT mice. However, the expression level of ACE2 protein was decreased and that of TACE was increased in the kidneys of *Col4a3*^−/−^ mice compared with those of WT mice. Ang-[1–7] treatment recovered the levels of ACE2 and TACE. Collectively, these findings demonstrate that deterioration of ACE2/Ang- [1–7]/Mas receptor axis were prominent in the kidneys of *Col4a3*^−/−^ mice, but that these changes are effectively blocked by Ang-[1–7] treatment. Since a recent study showed that mitogen-activated protein kinase (MAPK) signaling affects ACE2 expression by regulating the activation of TACE^16^, we further performed immunoblot analysis for MAPK pathway members and their phosphorylated (activated) forms (Fig. 5). The levels of phosphorylated ERK and JNK were considerably enhanced in the kidneys of *Col4a3*^−/−^ mice compared with those of WT mice, which were attenuated by Ang-[1–7]. These results suggest that ERK and JNK MAPK signaling might play a role in the TACE-induced down-regulation of ACE2 in the kidneys of *Col4a3*^−/−^ mice.

### Effects of Ang-[1–7] on fibrosis, inflammation and apoptosis in TGF-β-stimulated HK-2 cells

**Figure 6:**
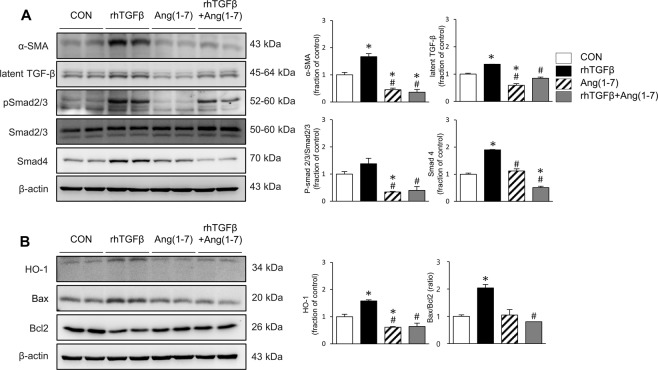
Effects of Ang-[1–7] on fibrosis, inflammation and apoptosis in TGF-β treated HK-2 cell. (**A**) Comparison of expression level for fibrosis markers and Smad proteins determined by immunoblotting in HK-2 after stimulation with vehicles or recombinant human TGF-β (rhTGFβ). (**B**) Comparison of expression level for inflammation and apoptosis proteins determined by immunoblotting in HK-2 after stimulation with vehicles or rhTGFβ. β-actin was used as the endogenous control. Each column represents mean ± SEM. *P < 0.05 vs. control (CON) group; ^#^P < 0.05 vs^.^ rhTGFβ group.

**Figure 7:**
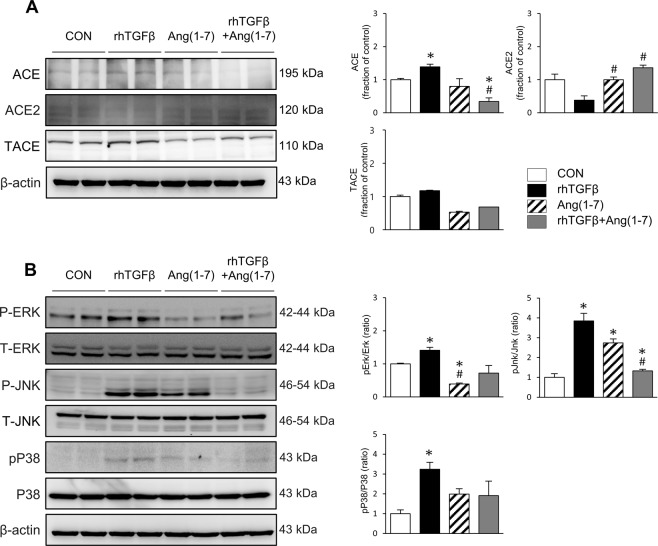
Effects of Ang-[1–7] on RAS and MAPK pathway in TGF-β treated HK-2 cell. Comparison of expression level for RAS (**A**) and MAPK pathway proteins (**B**) determined by immunoblotting in HK-2 after stimulation with vehicles or recombinant human TGF-β (rhTGFβ). β-actin was used as the endogenous control. Each column represents mean ± SEM. *P < 0.05 vs. control (CON) group; ^#^P < 0.05 vs. rhTGFβ group.

Finally, we conducted *in vitro* studies in the human proximal tubular epithelial cell line HK-2 to further explore the effects of Ang-[1–7] on the pro-fibrotic condition at the cellular level. HK-2 cells were stimulated by TGF-β, a critical mediator of renal fibrosis (Fig. 6A), resulting in significant increases in the phosphorylation of Smad2/3 and expression of Smad 4, which are downstream signals of TGF-β, along with increased expression levels of α-SMA. However, the induction of these profibrotic proteins was blocked by Ang-[1–7] treatment. Similarly, TGF-β enhanced HO-1 expression, which was ameliorated by Ang-[1–7] co-treatment (Fig. 6B). The BAX/BCL-2 ratio was increased by TGF-β treatment, which was also recovered by Ang-[1–7] cotreatment, indicating protection against apoptosis (Fig. 6B). TGF-β treatment further increased the levels of ACE, and decreased the level of ACE2 (Fig. 7A). After cotreatment with Ang-[1–7], ACE levels considerably diminished, and ACE2 expression recovered. TGF-β-induced ERK, JNK, and p38 phosphorylation was also suppressed by Ang-[1–7] co-treatment, although a significant reduction was only observed for phosphorylated JNK (Fig. 7B). These findings in TGF-β-stimulated human cells corresponded with the results in *Col4a3*^−/−^ mouse kidneys, demonstrating a common mechanism of Ang-[1–7] renal protection.

## Discussion

The present results clearly demonstrate that Ang-[1–7] could ameliorate the renal injury in *Col4a3*^−/−^ mice as an experimental model of AS. Specifically, Ang-[1–7] effectively attenuated fibrosis, inflammation, and apoptosis in the kidneys of *Col4a3*^−/−^ mice, and protected against TGF-β-induced cellular injury in human proximal tubular epithelial cells. These findings highlight the potential of Ang-[1–7] as a therapeutic agent not only for AS but also for CKD.

Ang-[1–7] is a peptide produced from Ang II by ACE2, Ang-[1–9] by ACE or Ang I by endopeptidase such as neprilysin^17^. Ang-[1–7] has well-established cardioprotective and renoprotective effects due to its anti-fibrosis and anti-inflammatory potential^18^. Indeed, in previous studies using other renal injury models such as models of diabetic nephropathy and obstructive nephropathy, Ang-[1–7] showed anti-inflammation, antioxidant, and anti-fibrosis effects^4,19^. In studies using rat proximal tubule NRK-52 cells, Ang-[1–7] abolished advanced glycated end product-induced cellular hypertrophy and myofibroblast transformation via inhibition of ERK MAPK^20^. Similarly, we demonstrated that Ang-[1–7] ameliorated the severe glomerulosclerosis, and interstitial inflammatory cell infiltration and accumulation of extracellular matrix in the kidneys of *Col4a3*^−/−^ mice. Further, knockout of the *Col4a3* gene led to the marked upregulation of fibrosis markers such as α-SMA and fibronectin, which was suppressed by Ang-[1–7] treatment, indicating protection against renal fibrosis at the gene transcription level. TGF-β is a critical mediator of kidney fibrosis and is known to be associated with disease progression in both AS patients and *Col4a3*^−/−^ mice^21^. We confirmed the increase in latent TGF-β expression and activation of Smad signaling in *Col4a3*^−/−^ mouse kidneys and human HK-2 cells, which was inactivated by Ang-[1–7] treatment. Taken together, these data suggest that Ang-[1–7] ameliorates kidney fibrosis in *Col4a3*^−/−^ mice. As tissue fibrosis is the common pathophysiologic process of progressive renal disease from various causes^22^, Ang-[1–7] might be a candidate for development of a therapeutic peptide that inhibits CKD progression. Intrarenal inflammation and podocyte apoptosis are also prominent features in AS^23,24^, and we also demonstrated that Ang-[1–7] effectively suppressed inflammation, apoptosis, and oxidative stress in the kidneys of *Col4a3*^−/−^ mice, further demonstrating a protective role against renal damage in progressive disease.

Dysregulation of RAS is a well-established feature of experimental AS^13^, and previous studies with the *Col4a3*^−/−^ mouse model showed decreased tissue expression and plasma levels of Ang-[1–7] at 7 weeks of age, along with decreased intrarenal expression of ACE2, an enzyme involved in the production of Ang-[1–7], and fibrosis and inflammation of the kidneys were reduced with administration of murine recombinant ACE2^14^. Here, we have expanded on these previous findings by demonstrating that treatment with Ang-[1–7] itself can recover the dysregulated RAS in the kidneys of *Col4a3*^−/−^ mice, which upregulated the ACE2/Ang-[1–7]/Mas receptor axis. Since ACE/Ang II/AT1R axis hyperactivity is known to cause renal injury by accelerating renal fibrosis, inflammation, and oxidative stress, which is also found in AS, the ability of Ang-[1–7] administration to activate the ACE2/Ang-[1–7]/Mas receptor axis ultimately protected against renal injury in experimental AS.

We observed decreased expression of TACE and increased expression of ACE2 in the *Col4a3*^−/−^ mice kidney, and an improvement by the administration of Ang-[1–7]. TACE activity is known to induce cleavage and loss of tissue ACE2, thereby further exacerbating Ang II harmful effects. Such ACE2 regulation by TACE is caused by enzymatic cleavage, not by the regulation of gene transcription^16^. Therefore, uncoupling between tissue protein level and mRNA occurs. Previously, protein expression and activity of ACE2 were reduced in the 7-week-old *Col4a3*^−/−^ mice kidney, but mRNA level was not changed^13^. Unfortunately, our study did not check that mRNA levels of ACE2 did not change after Ang-[1–7] administration. Therefore, in our study, the decrease of ACE2 expression and recovery by Ang-[1–7] administration in the *Col4a3*^−/−^ mice kidney may be caused by TACE enzymatic cleavage, but also by various cytokines activated by TACE in the inflammation state.

Recently, Suh *et al*. reported about the effect of AT1R antagonist, on renal fibrosis in an experimental Alport syndrome model^25^. In that study, Suh *et al*. reported that olmesartan effectively suppressed the progression of tubulointerstitial fibrosis and inflammation in *Col4a3*^−/−^ mice, by interrupting RAS- TGF-β feedback loop to counterbalance intrarenal RAS activation. Olmesartan is one of the AT1R blocker class, which is known to have inhibitory effect on ACE^26^. Ishiyama *et al*. showed olmesartan increases Ang-[1–7] by inducing increased expression of ACE2^27^. Agata *et al*. observed that co-treatment of olmesartan and Ang-[1–7] antagonist significantly increased Ang II than that of treatment with olmesartan alone, which is indirectly showing that Ang-[1–7] contributes to Ang II suppression^28^. Taken together, olmesartan inhibits ACE and increases the expression of ACE2, increasing the production of Ang-[1–7]. Thus, we found that some of the effects of Olmesartan occur via Ang- [1–7], which may be the reason why the two drugs show similar effects.

Nevertheless, our research has some limitations. First, we did not measure the blood pressure (BP) of mice. However, other studies using similar doses of Ang-[1–7] did not reduce BP in the Ang II induced or high fat diet induced hypertension mice models^29–31^. Even the study conducted in our group used olmesartan instead of Ang-[1–7], which is generally expected to reduce BP, but did not reduce BP in *Col4a3*^−/−^ mice^25^. Nevertheless, a protective effect was observed. Ang-[1–7] is expected to have similar results and is thought to have a renoprotective effect regardless of BP. Second, previous studies have reported that Ang-[1–7] can act via receptors other than the Mas receptor^32^. Therefore, further *in vitro* studies using Mas antagonist may provide further insight into the renoprotective effects of Ang- [1–7] via the Mas receptor. Third, ACE2 treatment increased blood and kidney tissue levels of Ang- [1–7] in same AS model previously^14^. However, blood and kidney tissue levels of Ang- [1–7] was not measured in this study.

In conclusion, Ang-[1–7] presents anti-fibrotic, anti-inflammatory and anti-apoptotic action via recovery of altered RAS in experimental AS mice. Thus, Ang-[1–7] shows good potential as a treatment for inhibiting the progression of CKD.

## Methods

### Ethics statement

All experimental methods were performed in accordance with the relevant guidelines and regulations. The experimental protocol was approved by the Animal Care Regulations Committee of Chonnam National University Medical School (CNU IACUC-H-2018-22).

### Experimental animals and protocols

Wild-type (WT) and *Col4a3*^−/−^ mice on a congenic 129X1/SvJ background were purchased from the Jackson Laboratory (Bar Harbor, ME, USA), and only male mice were used in this study. The mice were housed at the animal care facility at the Chonnam national university medical school, and fed mice standard diet with ad libitum access to water. We only used male mice in this study. Tail tip genotyping was performed to verify the genotype for *Col4a3*^−/−^ mice by using the following primers: common, 5′-CCA GGC TTA AAG GGA AAT CC-3′; WT reverse, 5′-TGC TCT CTC AAA TGC ACC AG-3′; and mutant reverse, 5′-GCT ATC AGG ACA TAG CGT TGG-3′.

We treated *Col4a3*^−/−^ mice with Ang-[1–7] (H-1715; Bachem Americas, Torrance, CA, USA) after weaning from 4 to 7 weeks of age, before mortality became a significant confounding factor. For Ang-[1–7] treatment studies, the following groups of mice were studied beginning at 4 weeks of age for a period of 3 weeks via subcutaneously implanted micro-osmotic pump (model 1004; Alzet Osmotic Pumps, Cupertino, CA, USA): (i) Col4a3^−/−^ mice that received Ang-[1–7] at a dose of 25 μg/kg/hour, (ii) Col4a3^−/−^ mice that received saline, and (iii) wild-type littermate controls that received saline (n = 4 mice/group). The dose of Ang-[1–7] was generally used dose according to previous reports^29–31,33^. The *in vivo* experiment was repeated in two sets to see reproducibility.

### Urinary NGAL and microalbuminuria measurement

Urine of mice was collected by maintaining mice in individual metabolic cages for the last 3 days of the experiment. Mice were maintained in individual metabolic cages for the last 3 days of the experiment to allow urine collection. Urine samples were centrifuged at 8000 g for 5 minutes immediately after collection. Urinary level of NGAL were measured with a commercial ELISA kit (R&D Systems, Minneapolis, MN, USA), according to manufacturer’s instruction. Urinary microalbumin was measured using the turbidimetric immunoassay method (Olympus AU 5431, Toshiba TBA-200FR autoanalyzer, Tokyo, Japan), while urinary creatinine was measured using the Jaffe method. Urinary albumin excretion was estimated as the albumin-to-creatinine ratio in milligrams of albumin per gram of creatinine.

### Semiquantitative immunoblotting

Western blot analysis was performed as previously described^4,34^. Kidney tissues were homogenized in ice-cold isolation solution containing 0.3 M sucrose, 25 mM imidazole, 1 mM ethylenediamine tetraacetic acid (EDTA), 8.5 mM leupeptin, and 1 mM phenylmethylsulfonyl fluoride (pH 7.2). The homogenates were centrifuged at 4000 × g for 15 minutes at 4 °C to remove whole cells, nuclei, and mitochondria. The total protein concentration was measured by bicinchoninic acid (BCA) assay kit (Pierce, Rockford, IL, USA). All samples were adjusted to reach the same final protein concentrations. They were then dissolved at 65 °C for 15 minutes in SDS-containing sample buffer and stored at −20 °C. To confirm equal loading of proteins, an initial gel was stained with Coomassie blue. SDS-PAGE was performed on 9 or 12% polyacrylamide gels. The proteins were electrophoretically transferred onto nitrocellulose membranes (Hybond ECL RPN3032D; Amersham Pharmacia Biotech; Little Chalfont, UK) using Bio-Rad Mini Protean II apparatus (Bio-Rad; Hercules, CA, USA). The blots were blocked with 5% milk in PBS-T (80 mM Na_2_HPO_4_, 20 mM NaH_2_PO_4_, 100 mM NaCl, and 0.1% Tween-20 at pH 7.5) for 1 hour; incubated overnight at 4 °C with primary antibodies; and incubated with secondary anti-rabbit, anti-mouse, or anti-goat horseradish peroxidase-conjugated antibodies thereafter. The immunoblots were then visualized using an enhanced chemiluminescence system. Protein levels were quantified using densitometry. The relative intensities of immunoblot signals were measured by densitometry using Scion image for windows software (Scion Corporation, 2000–2001. version Alpha 4.0.3.2. MD, USA) and were expressed as fold changes relative to control. Primary and secondary antibodies used in immunoblottings are listed in Table S1.

### Real-time polymerase chain reaction (Real-Time PCR)

Polymerase chain reaction analysis was performed as previously described^35^. Renal cortex was homogenized in Trizol reagent (Invitrogen, Carlsbad, CA, USA). RNA was extracted with chloroform, precipitated with isopropanol, washed with 75% ethanol, and then dissolved in distilled water. The RNA concentration was determined by the absorbance read at 260 nm (Ultraspec 2000; Pharmacia Biotech, Cambridge, UK). The mRNA expression of inflammatory cytokines and adhesion molecules was determined by real-time PCR. cDNA was made by reverse transcribing 5 μg of total RNA using oligo (dT) priming and superscript reverse transcriptase II (Invitrogen, Carlsbad, CA, USA). cDNA was quantified using Smart Cycler II System (Cepheid, Sunnyvale, CA, USA) and SYBR Green was used for detection. Each PCR reaction was done in 10 pM forward primer, 10 pM reverse primer, 2X SYBR Green Premix Ex Taq (TAKARA BIO INC, Seta 3-4-1, Japan), 0.5 μl cDNA and H2O to bring the final volume to 20 μl. Relative levels of mRNA were determined by real-time PCR, using a Rotor-GeneTM 3000 Detector System (Corbette research, Mortlake, NSW, Australia). Sequences of primers are listed in Table S2.

The PCR was performed according to the following steps: (1) 95 °C for 5 minutes; (2) 95 °C for 20 seconds; (3) 58 to 60 °C for 20 seconds (optimized for each primer pair); (4) 72 °C for 30 seconds to detect SYBR Green. Steps 2–4 were repeated for additional 40 cycles, while at the end of the last cycle temperature was increased from 60 to 95 °C to produce a melt curve. Data from the reaction were collected and analyzed with the Corbett Research Software. The comparative critical threshold values from quadruplicate measurements were used to calculate the gene expression, with normalization to GAPDH as an internal control. Melting curve analysis was performed to enhance specificity of the amplification reaction.

### Histology

Preparation and staining of the kidney tissue proceeded as previously described^35^. Kidney tissues were fixed with 4% paraformaldehyde, embedded in paraffin, and cut into 3 μm-thick sections. Hematoxylin and eosin (H&E) staining was performed to assess the histological morphology. The kidney tissue section slides were incubated in Gill’s hematoxylin for 5 min, washed with tap water, incubated in 95% ethanol, and stained with eosin and phloxine for 1 min. Subsequently, the sections were dehydrated in ethanol and xylene, and were mounted with Canada balsam. For Masson’s trichrome staining, after deparaffinization with xylene, the sections were treated with Bouin’s solution at 56 °C for 30 min and were washed under running tap water until the sections were clear. The sections were subsequently stained with Weigert’s hematoxylin, followed by staining with Biebrich Scarlet/Acid Fuchsin solution for 10 min and washing with distilled water. The sections were incubated with phosphotungstic acid/phosphomolybdic acid solution for 10 min and were treated with Aniline Blue solution for 15 min. They were subsequently incubated with acetic acid for 1 min and were dehydrated with ethanol and xylene. Collagen depositions, nuclei, and muscle fibers were stained blue, black, and red, respectively. Primary and secondary antibodies used in immunohistochemistry are listed in Table S3. Apoptosis of tubular epithelial cells was detected with TUNEL staining with ApopTag Plus Peroxidase *In Situ* Apoptosis Kit (Sigma-Aldrich), according to the manufacturer’s instruction.

### Cell culture and reagents

*In vitro* studies were conducted as previously described^35^. In short, human renal proximal tubular epithelial cells (HK-2 cells, American Type Culture Collection, Manassas, VA, USA) were used for *in vitro* study. HK-2 cells were cultured and passaged every 3~4 days in 100-mm dishes containing combined Dulbecco’s modified Eagle’s (DMEM) and Hams F-12 medium (Welgene, Daegu, Korea) supplemented with 10% fetal bovine serum (FBS; Welgene), 100 U/ml penicillin, and 100 mg/ml streptomycin (Sigma-Aldrich, St. Louis, MO, USA). HK-2 cells were then incubated in a humidified atmosphere of 5% CO_2_ and 95% air at 37 °C for 24 h, and sub-cultured until 70–80% confluence. HK-2 Cells were plated onto 60-mm dishes in a medium containing 10% FBS and incubated for 24 hours. The cells were then incubated in DMEM-F12 medium with serum free FBS and treated with rhTGFβ (2 ng/ml; R&D Systems) for an additional 16 hours. Ang-[1–7] was added 1 hours prior to rhTGFβ treatment. All the *in vitro* experiments were repeated in two sets to see reproducibility and done within the 30th passage of cells.

### Statistical analysis

The results were expressed as mean ± standard error of the mean (SEM). Multiple comparisons among the 3 groups were performed using one-way analysis of variance (ANOVA) and the *post-hoc* Tukey’s honestly significant difference test. Differences with values of p < 0.05 were considered significant.

## Supplementary information

Supplementary information.
