# Supplementary material for: Angiotensin-[1–7] attenuates kidney injury in experimental Alport syndrome
Source: Sci Rep. 2020 Mar 6;10:4225. doi: 10.1038/s41598-020-61250-5 (PMC7060323; doi:10.1038/s41598-020-61250-5)

Table S1. List of primary and secondary antibodies for immunoblotting

	Host	Reactivity	Supplier	Cat. No.
Angiotensin-converting enzyme	Goat	Human, Mouse	Santa Cruz	sc12187
Angiotensin-converting enzyme 2	Goat	Mouse	R&D	AF3437
Angiotensin-converting enzyme 2	Rabbit	Human	Cell Signaling	#4355
Bax	Rabbit	Human, Mouse	Cell signaling	#2772
Bcl-2	Rabbit	Human, Mouse	Cell signaling	#3498
ED-1	Rabbit	Mouse	Abcam	ab31630
Caspase 3	Rabbit	Human, Mouse	Cell signaling	#9662
Cleaved caspase 3	Rabbit	Human, Mouse	Cell signaling	#9661
Erk1/2	Rabbit	Human, Mouse	Cell signaling	#9102
Fibronectin	Rabbit	Human, Mouse	Abcam	ab2413
Heme oxygenase 1	Mouse	Mouse	Abcam	ab13248
JNK	Rabbit	Human, Mouse	Cell signaling	#9252
p38	Rabbit	Human, Mouse	Cell signaling	#9212
Phopho-Erk1/2	Rabbit	Human, Mouse	Cell signaling	#9101
Phopho JNK	Rabbit	Human, Mouse	Cell signaling	#9251
Phospho p38	Rabbit	Human, Mouse	Cell signaling	#9215
Phospho Smad2/3	Rabbit	Human, Mouse	Cell Signaling	#8828
Smad4	Rabbit	Human, Mouse	Cell Signaling	#38454
Smad6	Rabbit	Human, Mouse	Abcam	Ab13727
Smad2/3	Rabbit	Human, Mouse	Cell Signaling	#3012
TNFα-converting enzyme (TACE)	Rabbit	Human, Mouse	Millipore	AB19027
Transforming growth factor β	Rabbit	Human, Mouse	Cell Signaling	#3711
α smooth muscle actin	Mouse	Human, Mouse	Sigma-Aldrich	A3854
β-actin	Rabbit	Human, Mouse	Cell Signaling	#3711

Goat IgG, HRP-linked	Rabbit	Goat IgG	Sigma-Aldrich	AP106P
Rabbit IgG, HRP-linked	Goat	Rabbit IgG	Cell Signaling	#7074
Mouse IgG, HRP-linked	Horse	Mouse IgG	Cell Signaling	#7076

Table S2. List of primer sequences for real-time qPCR

	Forward	Reverse
mActa2 (αSMA)	ACTGGGACGACATGGAAAAG	CATCTCCAGAGTCCAGCACA
mCol1a1 (collagen, type I)	GAGCGGAGAGTACTGGATCG	TACTCGAACGGGAATCCATC
mGapdh	TGTGTCCGTCGTGGATCTGA	GATGCCTGCTTCACCACCTT
mIcam1	AACTTTTCAGCTCCGGTCCTG	TCAGTGTGAATTGGACCTGCG
mCcl2 (MCP-1)	ATCCCAATGAGTAGGCTGGAGAGC	CAGAAGTGCTTGAGGTGGTTGTG
mFn1 (Fibronectin)	ACACGGTTTCCCATTACGCCAT	AATGACCACTGCCAAAGCCCAA
mTgfb1 (TGFβ)	CAACAATTCCTGGCGTTACCTTGG	GAAAGCCCTGTATTCCGTCTCCTT
mTnf (TNFα)	GCATGATCCGCGACGTGGAA	AGATCCATGCCGTTGGCCAG
Vcam1	TCTCTCAGGAAATGCCACCC	CACAGCCAATAGCAGCACAC

Table S3. List of primary and secondary antibodies for immunohistochemistry

	Host	Reactivity	Supplier	Cat. No.
Collagen type 1	Rabbit	Mouse	Abcam	ab34710
F4/80	Rat	Mouse	Bio-rad	MCA497GA
Transforming growth factor β1	Rabbit	Human, Mouse	Abcam	ab92486
α smooth muscle actin	Mouse	Human, Mouse	Sigma-Aldrich	A3854
Rabbit IgG, HRP-linked	Goat	Rabbit IgG	Vector	PI-1000
Rat IgG, HRP-linked	Goat	Rat IgG	Vector	PI-9400
Mouse IgG, HRP-linked	Goat	Mouse IgG	Vector	PI-2000

Related to Figure 1

B

αSMA 43 kDa

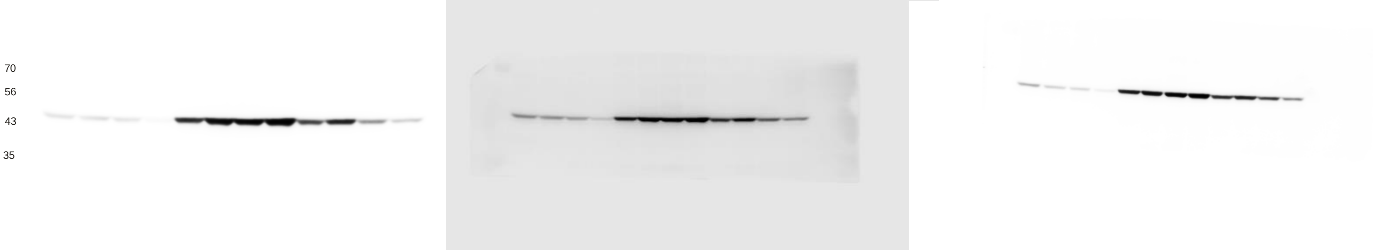

FN 240 kDa

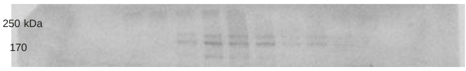

Original image of FN

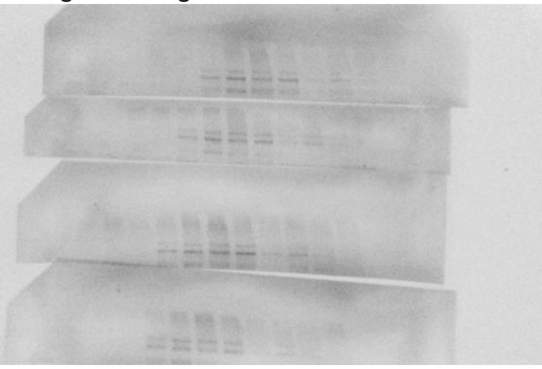

The anti-fibronectin antibody (abcam ab2413) we used for immunoblotting detection marks fibronectin at 240KDa. Therefore, GenDEPOT P8502-050 (6-240kDa) was used as a protein marker. After the membrane transfer process, fibronectin (240kDa) was detected by cutting more than 170kDa, and other proteins with relatively small MW (p-Smad2/3, TGFβ) were detected in the membrane below 130kDa. The following figures are the corresponding membranes.

Psmad2/3

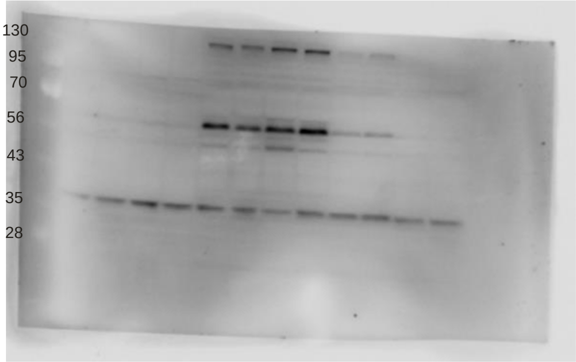

TGF-b

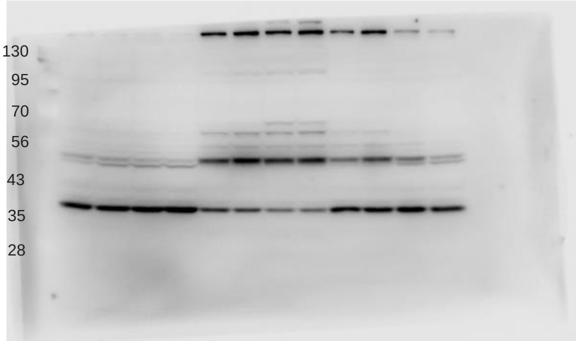

TGF-b

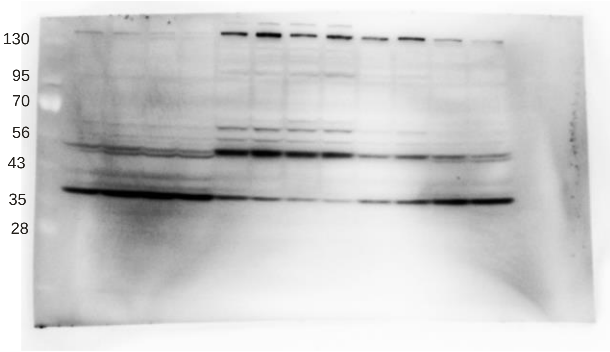

β-actin 43 kDa

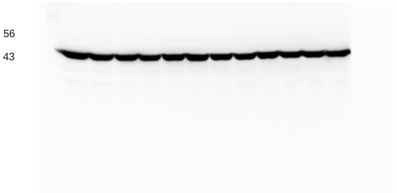

Related to Figure 2

A

TGFβ 45-64 kDa

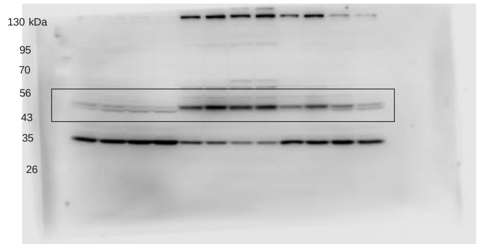

pSMAD2/3 52-60 kDa

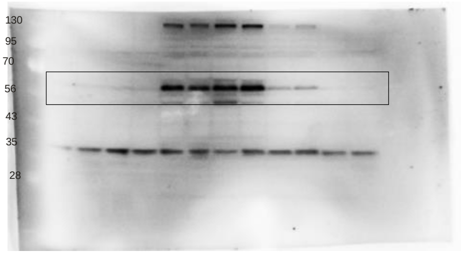

SMAD2/3 50-60 kDa

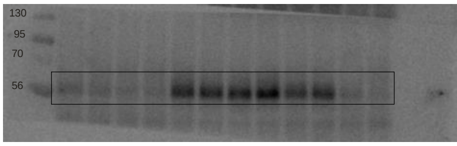

Smad6 52kDa

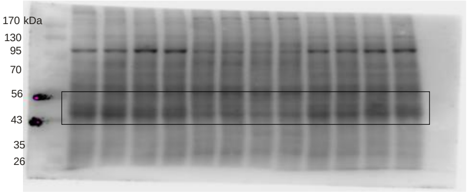

SMAD4 70 kDa

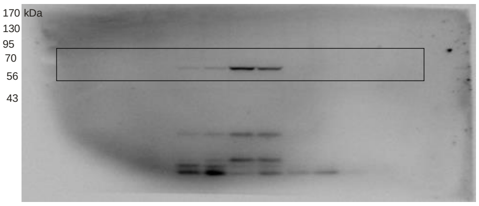

β-actin 43 kDa

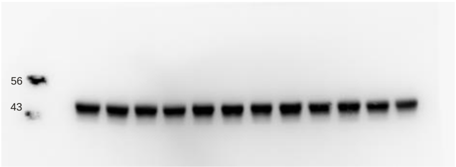

Related to Figure 3

A

CD68 (ED-1) 37 kDa

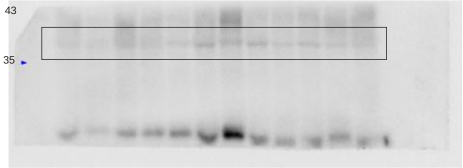

HO-1 34 kDa

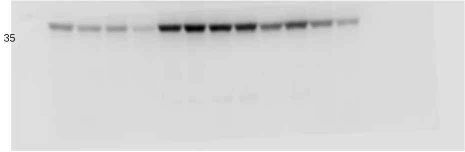

β-actin 43 kDa

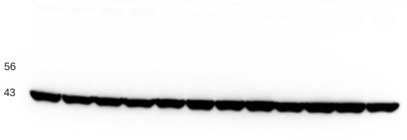

C

Cleaved caspase3 17-19 kDa

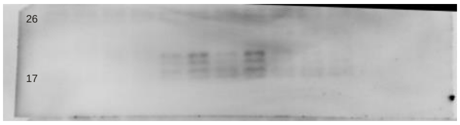

Caspase3 35 kDa

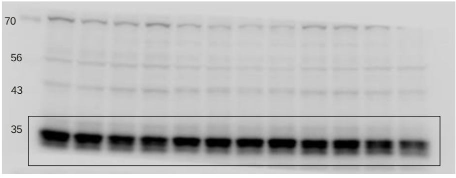

BAX 20 kDa

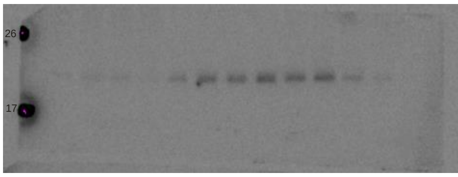

BCL2 26 kDa

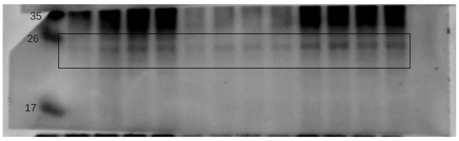

β-actin 43 kDa

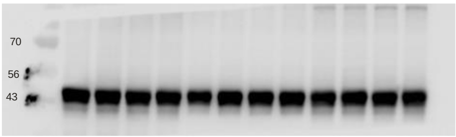

Related to Figure 4

ACE 195 kDa

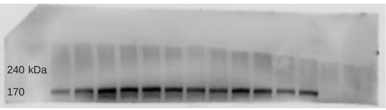

Original image of ACE

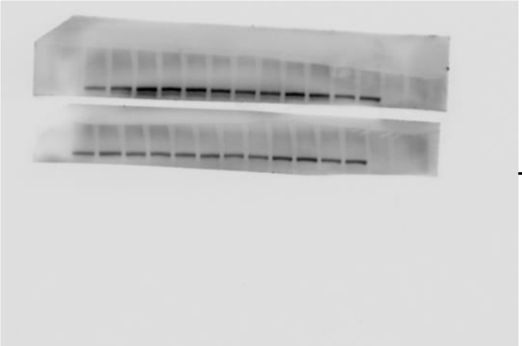

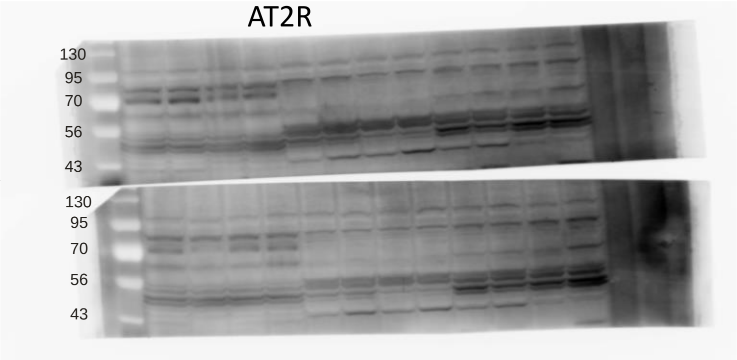

The anti-ACE antibody (Santa Cruz sc12187) we used for immunoblotting detection marks ACE at 195 KDa. Therefore, GenDEPOT P8502-050 (6-240 kDa) was used as a protein marker. After the membrane transfer process, ACE (195kDa) was detected by cutting more than 170kDa, and other proteins with relatively small MW (AT2R) were detected in the membrane below 130kDa. The following figures are the corresponding membranes.

TACE 110 kDa

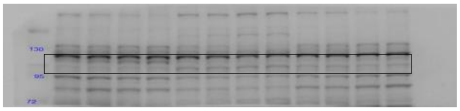

Original image of TACE

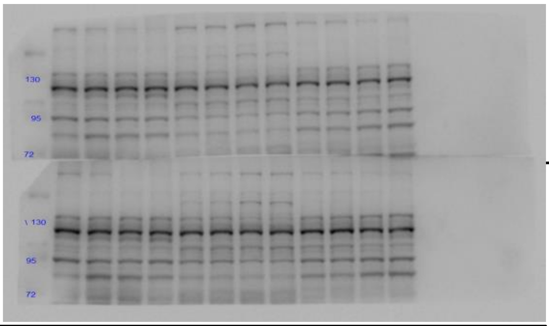

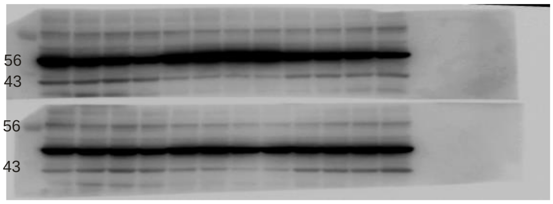

The anti-TACE antibody (Millipore ab19027) we used for immunoblotting detection marks TACE at 110 KDa. After the membrane transfer process, TACE (110kDa) was detected by cutting more than 72kDa, and other proteins with relatively small MW (not included in figure) were detected in the membrane below 72kDa.
The following figures are the corresponding membranes.

ACE2 120 kDa

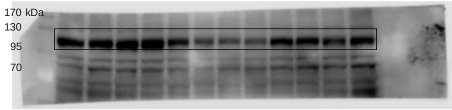

β-actin 43 kDa

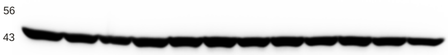

Related to Figure 5

pERK 42-44 kDa

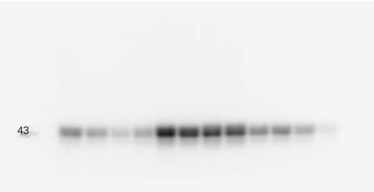

ERK 42-44 kDa

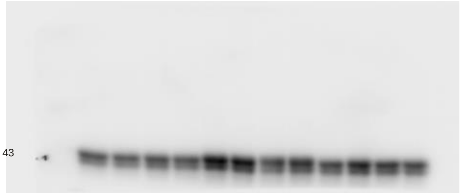

pJNK 46-54 kDa

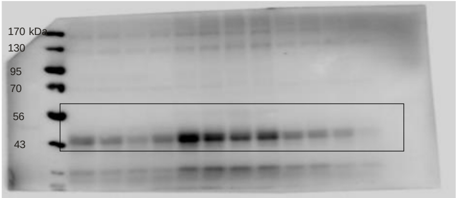

JNK 46-54 kDa

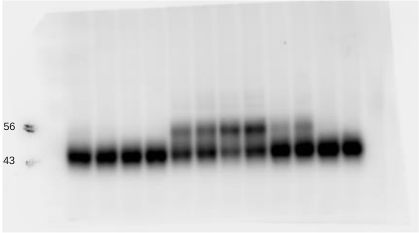

Overexpression of JNK membrane

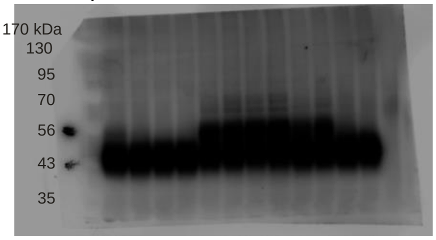

The two figures on the left are the same membrane. The marker was not well identified in the original image, so it was overexpressed on purpose and the image was detected by whole gel.

pP38 43 kDa

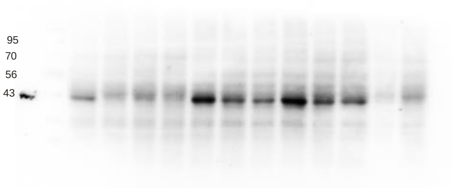

P38 43 kDa

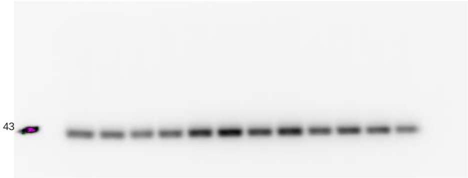

β-actin 43 kDa

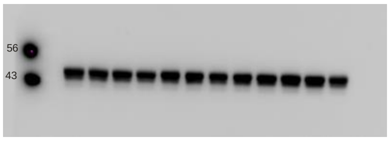

Related to Figure 6

A

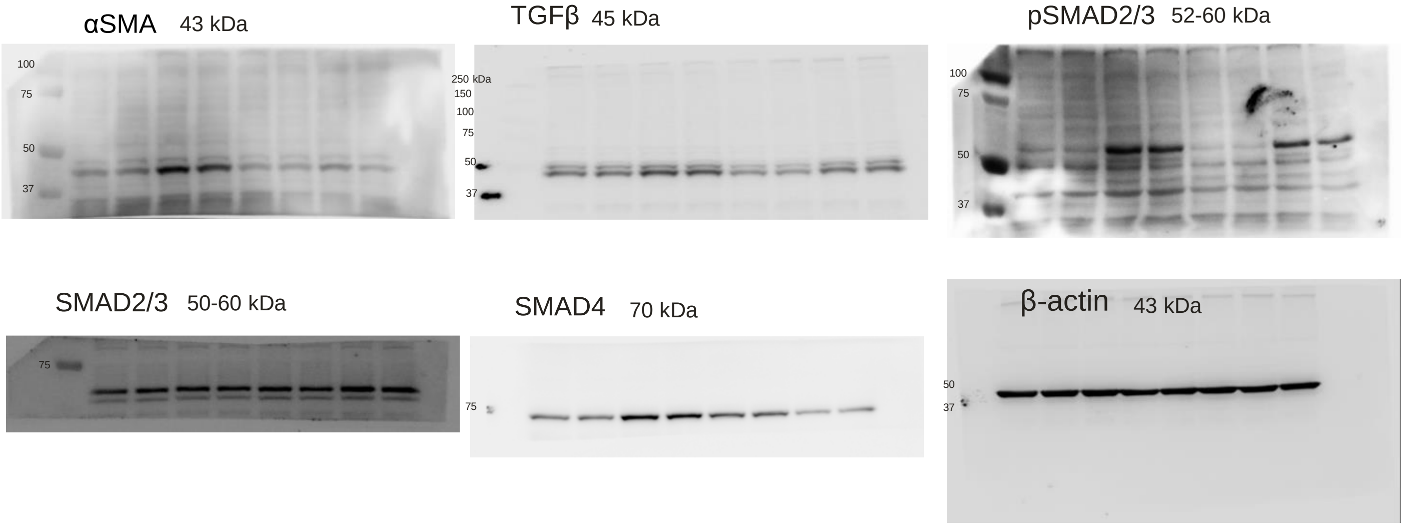

B

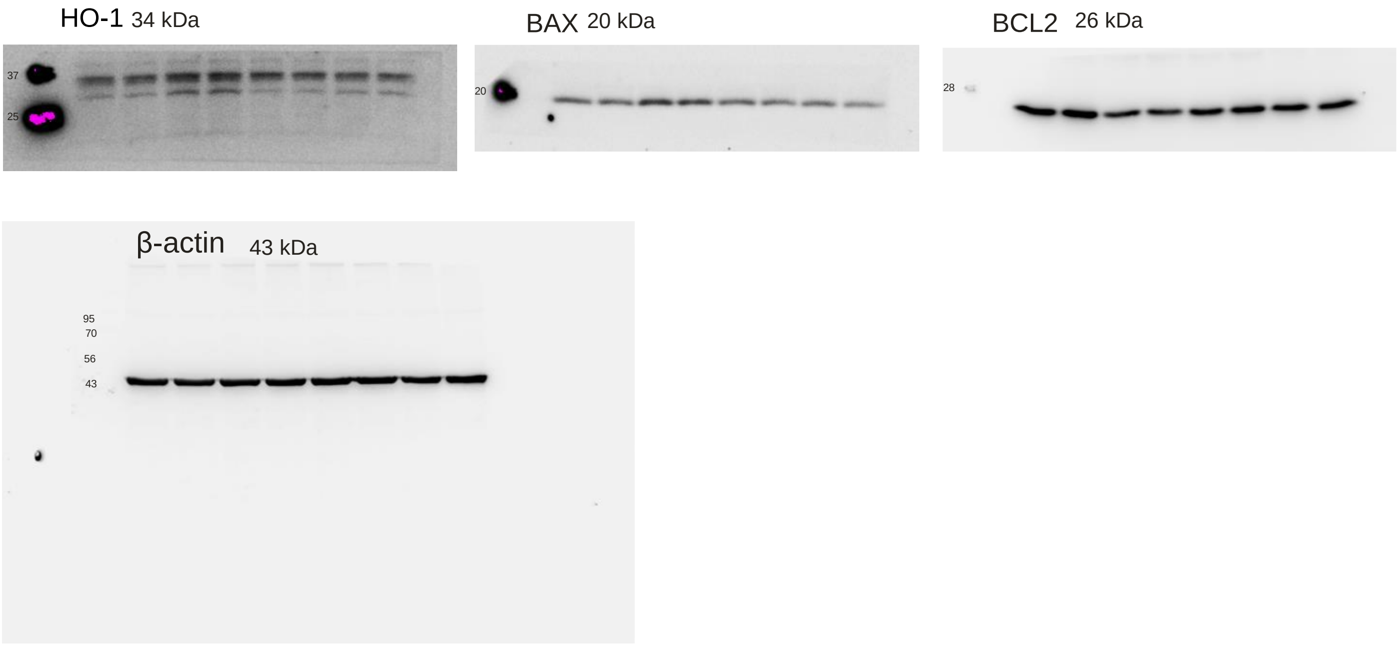

Related to Figure 7

A

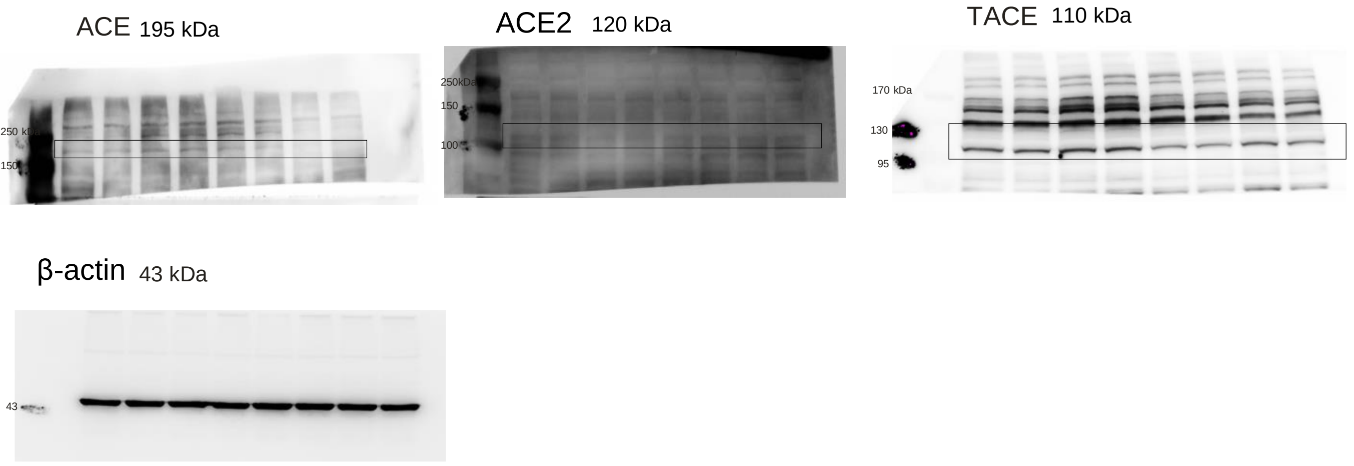

B

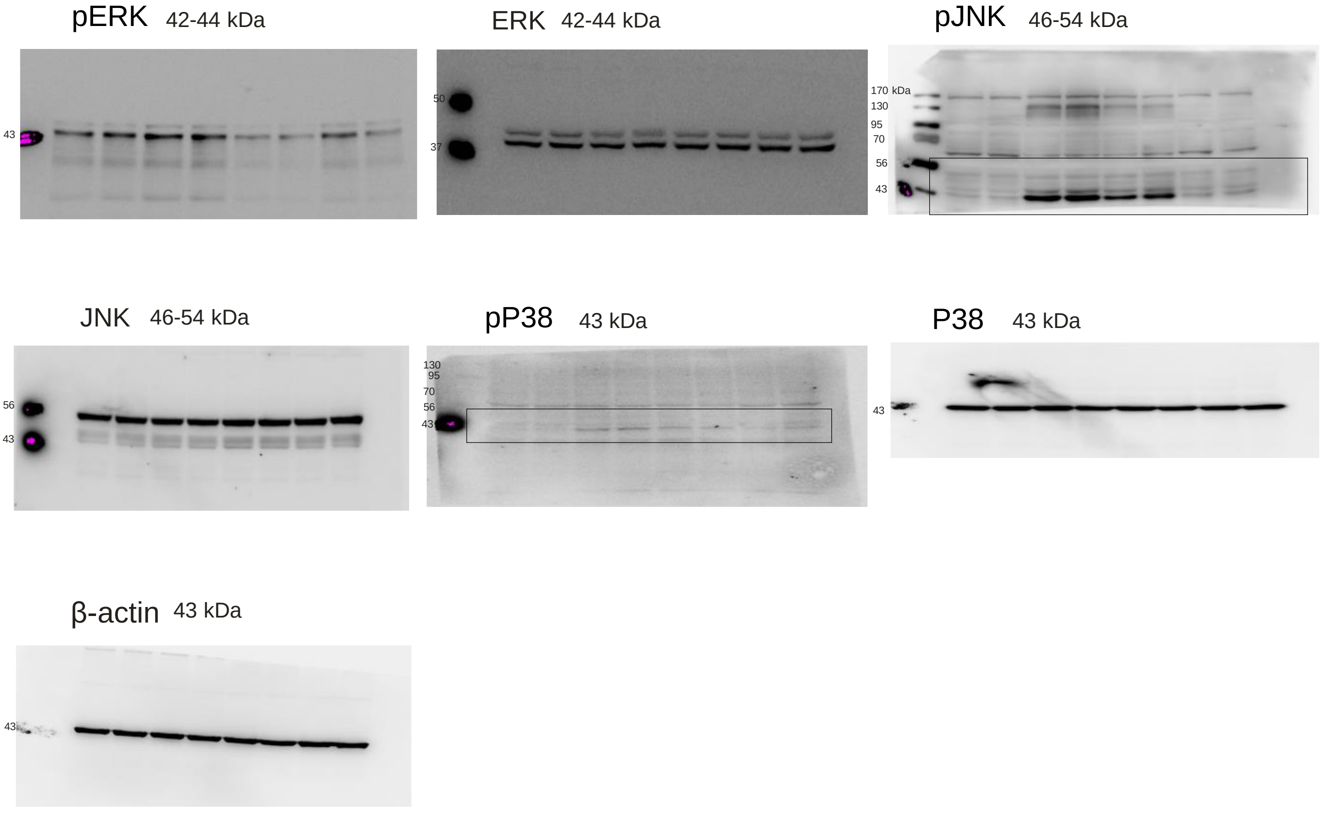

Supplement: Supplementary file 1 — Supplementary information. [file 41598_2020_61250_MOESM1_ESM.pdf]
